# Effects of Length-to-Width Ratio on Magnetic and Microstructural Properties of Die-Upset Nd–Fe–B Magnets

**DOI:** 10.3390/ma17215236

**Published:** 2024-10-28

**Authors:** Sang-Hyub Lee, Min-Suk Kang, Yang-Do Kim, Dong-Hwan Kim

**Affiliations:** 1 R&D Center, Star Group Ind. Co., Ltd., Daegu 42714, Republic of Korea; leesh@sgtech.co.kr (S.-H.L.); mskang@sgtech.co.kr (M.-S.K.); 2 Department of Materials Science and Engineering, Pusan National University, Busan 46241, Republic of Korea

**Keywords:** Nd–Fe–B magnet, die-upset magnet, net-shaped magnet, grain refinement

## Abstract

In this study, the effects of the length-to-width ratio on the magnetic and microstructural properties of die-upset Nd–Fe–B magnets were examined. A die-upset magnet with a uniform shape and no significant cracking was successfully developed. During the die-upset process, the applied pressure was not uniform across the magnet and varied depending on its shape and position. In the case of the magnet with a 4:1 ratio, which had the largest length-to-width ratio, there was a higher concentration of stress at the edges compared to the center, resulting in easier grain deformation and growth at the edges, which led to the lowest remanence and (*BH*)_max_. As the length-to-width ratio approached 1:1, the grain size increased slightly, reducing the coercivity; however, the magnets maintained a uniform grain-size distribution. The change from a rectangular to a square pressing surface resulted in a more uniform stress distribution, particularly at the center and corners, which improved the consistency of the magnetic properties across different regions of the magnet. Consequently, an 11.9% enhancement in (*BH*)_max_, reaching 285.6 kJ/m^3^, was achieved in the 1:1 ratio die-upset magnet, which is attributed to the uniform grain size and improved grain alignment.

## 1. Introduction

Nd–Fe–B magnets are widely used in various high-performance applications, including electric vehicles, wind turbines, and other energy-efficient devices, owing to their excellent magnetic properties, such as high remanence and coercivity [1,2]. The addition of heavy rare-earth elements (HREEs) such as Dy and Tb is a common practice for enhancing the coercivity of these magnets, particularly for high-temperature applications [1,2,3]. While these HREEs improve coercivity, they have significant drawbacks, including high costs, limited availability, and negative environmental impacts. Anisotropic hot-deformed magnets have finer microstructures (200–500 nm), which are an order of magnitude smaller than those of sintered magnets [4,5,6]. Thus, it is easier to obtain high coercivity without the addition of HREEs compared with sintered Nd–Fe–B magnets. The thermal coefficient for hot-deformed magnets (~0.4%/°C) is superior to that for sintered magnets (~0.6%/°C), because the hot-deformed magnets have finer grain structures and more uniform grain alignment [7,8].

Anisotropic hot-deformed magnets are easily manufactured using the die-upset process, which utilizes high-density hot-pressed precursors [9,10,11]. However, achieving a near-net shape using this method presents challenges owing to issues such as nonuniform material flow, stress concentration, and grain structure [12,13,14,15]. During the die-upset process, the pressure is unevenly distributed, particularly for nonsymmetric shapes such as rectangles or squares, causing stress concentrations at the corners. This can lead to uneven deformation or cracking at the edges, as well as nonuniform grain growth [16]. Cracks formed at the edges must be completely removed, which requires substantial surface polishing. To reduce material waste, minimizing the extent of surface polishing is essential.

Dirba et al. reported the development of hot-deformed magnets without significant cracking using a die-upset process [16]. In their study, unlike the conventional die-upset method, where the material flows into an open cavity, the process was designed such that by the end of the hot deformation, the material fully reached the walls of the matrix. This approach resulted in homogeneous magnetic properties along the tablet radius. However, the magnets typically used in motors are elongated rectangular blocks, whereas the magnets reported had radial tablet shapes. Radial tablets must be cut and processed into rectangular blocks for motor applications [17]. The symmetric radial shape allows uniform pressure distribution during the die-upsetting process, mitigating stress concentration and the formation of cracks. In contrast, rectangular blocks are more practical for industrial use, but pose challenges in the die-upset process due to their uneven pressure distribution [16,18,19]. Additionally, we are facing limitations in the mass production and price competitiveness of die-upset magnets applicable to EV/HEV traction motors that surpass the characteristics of sintered magnets without using heavy rare-earth elements. To address this issue, the development of near-net-shape, block-form, rare-earth magnets, rather than the previously reported radial method, is necessary. However, in the closed die-upset method, stress concentration at the edges can lead to grain misalignment, resulting in differences in magnetic properties at different locations due to the stress variation between the center and edges of the magnet. Therefore, to develop near-net-shape, block-form, die-upset magnets, it is necessary to determine the optimal length-to-width ratio during the die-upset process.

Therefore, we investigated the effects of the length-to-width ratio on the magnetic and microstructural properties of block-shaped die-upset Nd–Fe–B magnets. Specifically, we examined how altering the length-to-width ratio from 4:1 to 1:1 affected the magnetic performance, focusing on key parameters such as the remanence, coercivity, and maximum energy product ((*BH*)_max_).

## 2. Experimental Procedures

**Figure 1:**
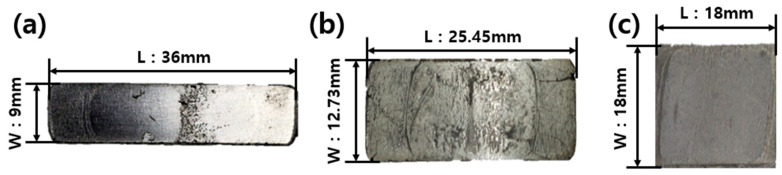
Visual comparison of Nd–Fe–B die-upset magnets with length-to-width ratios of (**a**) 4:1, (**b**) 2:1, and (**c**) 1:1.

An Nd–Fe–B die-upset magnet was fabricated using the die-upset technique. Melt-spun Nd–Fe–B flakes (MQU-F: Nd_13.6_Pr_0.2_Fe_75.6_Co_4.5_Ga_0.6_B_5.5_) were hot-pressed at 670 °C to obtain an almost fully dense and magnetically isotropic compact. The size of the hot-pressed (HP) magnet used for the die-upset process was 9 × 9 × 8 mm^3^. The compact was then die-upset at 735 °C in a vacuum with a 75% reduction in height using a closed die to prepare magnetically anisotropic magnets with a 4:1 ratio, whereas for samples with 2:1 and 1:1 ratios, the compact was die-upset at 765 °C. The closed die-upset process was employed, where the cavity size was varied to accommodate samples with different length-to-width ratios, while maintaining a constant thickness (2.2 mm) for all samples. This adjustment ensured that all samples had the same volume, despite the differences in shape. The cavity size was modified to reflect these variations in sample dimensions, and the process aimed to achieve uniform magnet volumes across different samples. Furthermore, the thickness of each sample was reduced by 75%, which indicates that the same pressure was applied during the die-upsetting process to achieve consistent magnet densities. Consequently, the only variation between the samples was their shape, as the volumetric and density parameters remained consistent. For clarity, refer to Table 1 for the specific dimensions and volume details of each sample. Additionally, Figure 1 provides a schematic representation of the die-upsetting process, where arrows and labels indicate the length and width for a clearer understanding of the dimensional changes.

To compare the magnetic properties and microstructures of different cross sections of the die-upset magnet, the magnet was cut using a wire saw. In the L-direction, the magnet with a 4:1 ratio was symmetrically divided into six sections around the center, the magnet with a 2:1 ratio was symmetrically divided into three sections around the center, and the magnet with a 1:1 ratio was symmetrically divided into two sections around the center. Additionally, all magnets were divided into three equal parts in the W-direction. The magnetic properties were measured with a B-H tracer (MAGNET-PHYSIK Permagraph C) and a vibrating sample magnetometer (VSM, Micro-Sense EZ 9 HF). For B-H tracer measurements, the specimens, which had been pre-saturated in the upward and downward pole directions (the thickness direction of the magnet), were aligned along the magnetization direction, and the magnetic field was applied for measurement. For VSM measurements, specimens were taken from the top center, top edge, center of the magnet, and center of the edge of each magnet. All samples were machined to similar sizes, aligned so that the magnetization direction (in the thickness direction of the magnet) was parallel to the applied magnetic field, and then measured. The magnetic flux per unit weight was calculated by measuring the magnetic flux using an electronic fluxmeter (MAGNET-PHYSIK EF5) and dividing it by the weight of the magnet. The uncertainty in the measurement of magnetic flux density was primarily influenced by the accuracy of the fluxmeter used, with an uncertainty of ±0.01 T. The fluxmeter was regularly calibrated to ensure precision. Additionally, environmental factors such as temperature fluctuations were minimized to reduce potential interference. For spatial positioning, the uncertainty was mainly due to alignment tolerances in positioning the sensor relative to the magnet. The positioning was controlled with a mechanical guide, and the uncertainty in spatial positioning was estimated to be within ±0.5 mm.

Microstructural observations and compositional analyses were performed using scanning electron microscopy (SEM). Average grain sizes and grain size distributions were evaluated by an image analyzer (UTHSCSA Image Tool 3.0.100). The orientation ratio (R; *I*_(006)_/*I*_(105)_) of the magnet was determined by comparing the (006) and (105) peaks in the X-ray diffraction (XRD) patterns.

## 3. Results and Discussion

Figure 1 shows the die-upset magnets manufactured with different length-to-width ratios. During the die-upset process, the HP magnet was set to flow toward the walls of the matrix, resulting in a smooth die-upset magnet with a uniform shape and no significant cracking. Although no external cracks were visually observed, minor surface processing was required. Surface polishing was performed on 5% of the magnet for analyzing the magnetic properties. Although the surfaces were not sufficiently clean for the magnets to be considered perfectly net-shaped, we developed crack-free rectangular magnets for traction motors of various sizes.

**Figure 2:**
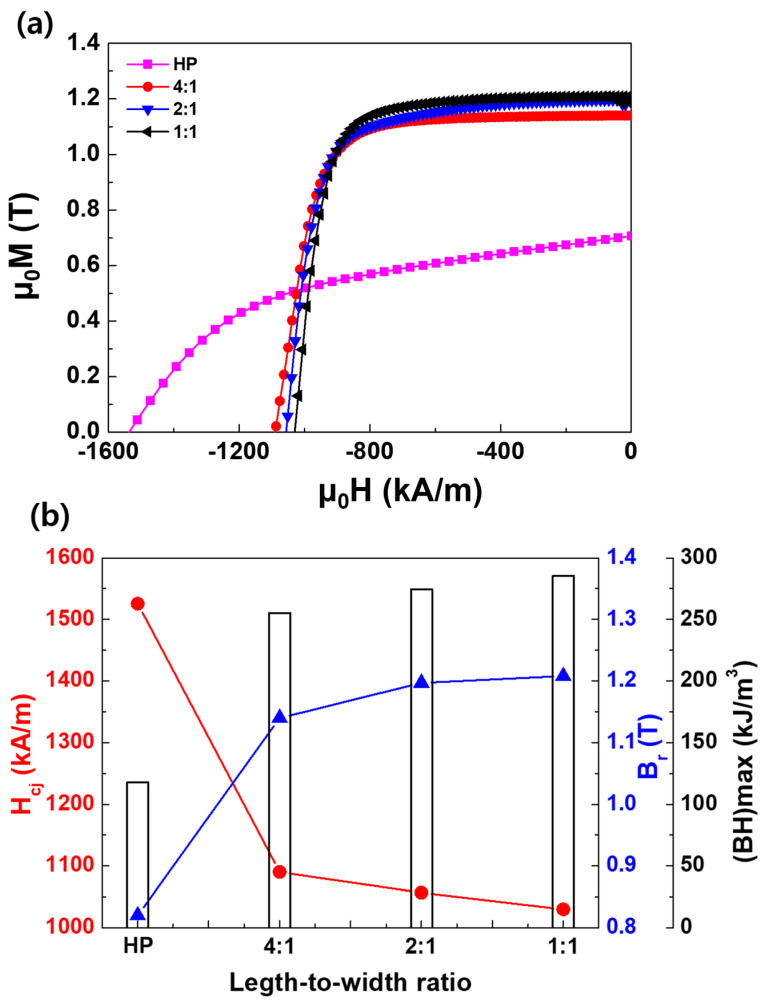
(**a**) Demagnetization curves and (**b**) magnetic properties of Nd–Fe–B HP and die-upset magnets with length-to-width ratios of 4:1, 2:1, and 1:1.

Figure 2a,b show the demagnetization curves and magntic properties of the HP magnet and the die-upset magnets with length-to-width ratios of 4:1, 2:1, and 1:1. The remanence and coercivity of the HP magnet used in the die-upset process were 1526 kA/m and 0.82 T, respectively. It is well known that the grain-boundary phase melts and anisotropic grain growth occurs during hot pressing [5,10]. The HP magnet has an isotropic structure, where the magnet is formed through pressing and heating, but the grains are not aligned in a specific direction. Since the grains are randomly oriented, the magnetization direction within the magnet was not uniform, resulting in a relatively low remanence value (Figure 2b). Anisotropic grain growth and crystal rotation, accompanied by grain-boundary sliding of the c-plane (the broad surface of the platelet-shaped grain), are promoted by stress during hot deformation [5,10]. This deformation step was key to achieving high degrees of texture and alignment, which increased the remanence. Therefore, the remanence of HP magnets had to be improved through a die-upset process. In the case of die-upset magnets, as the length-to-width ratio decreased (4:1 → 2:1 → 1:1), the coercivity decreased (1090 → 1057 → 1030 kA/m). After the die-upset process, the coercivity of all the magnets was significantly reduced compared with that of the HP magnets. There are several possible reasons for this. During the hot deformation process, residual stress may have formed because of the applied external stress [4,5]. This residual stress may have affected the movement of the domain walls, weakening their coercivity. However, the remanence and (*BH*)_max_ exhibited opposite trends. The remanence and (*BH*)_max_ of the magnets improved after the die-upset process, as shown in Figure 2b. The properties varied depending on the length-to-width ratio. Specifically, the remanence of the magnet with the 4:1 ratio was 1.14 T, while that of the magnet with the 2:1 ratio was increased to 1.20 T. A further reduction in the length-to-width ratio to 1:1 resulted in a slight increase in the remanence to 1.21 T. (*BH*)_max_ increased in the same manner as the remanence. This trend suggests that as the ratio approaches 1:1, the magnet becomes more anisotropic, potentially leading to better alignment of platelet-shaped grains and improved magnetic performance [5,10,11]. The coercivity of die-upset magnets relative to remanence can be improved through grain-boundary diffusion, heat treatment, and other post-processing methods; however, the remanence may decrease when such additional processes are applied [5,11]. Therefore, securing the remanence during the die-upset process is crucial. As the length-to-width ratio became closer to 1 (4:1 → 2:1 → 1:1), the remanence gradually improved while minimizing the reduction in coercivity.

**Figure 3:**
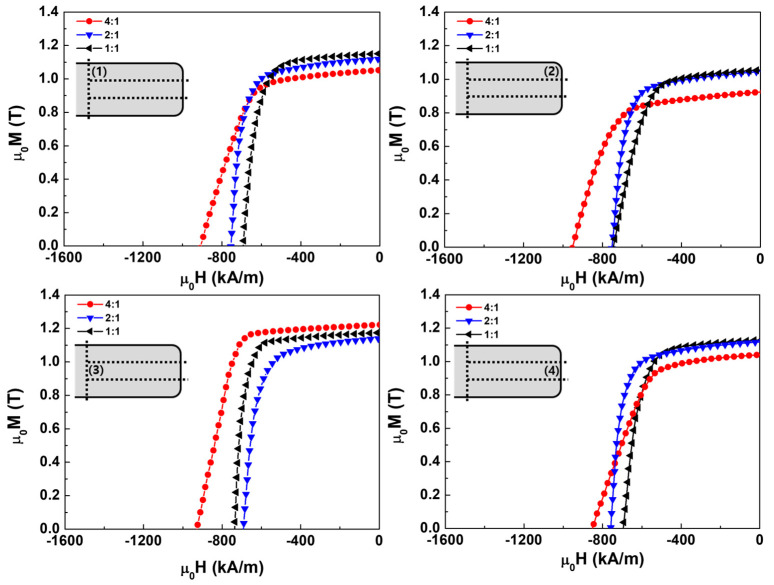
Demagnetization curves of die-upset magnets with length-to-width ratios of 4:1, 2:1, and 1:1, showing position-specific variations. The simplified illustration of a magnet included inside the graphs indicates the positions that were measured by VSM after cutting. The insets show the analysis positions: (**1**) top of each magnet’s center, (**2**) top of each magnet’s edge, (**3**) center of each magnet’s center, and (**4**) center of each magnet’s edge.

Figure 3 presents the demagnetization curves in different regions for die-upset magnets with ratios of 4:1, 2:1, and 1:1. The properties of the magnets are presented in Table 2. For the magnet with a 4:1 ratio, the coercivity did not vary significantly with respect to the position; however, the difference between the maximum (region 2) and minimum (region 4) coercivity regions was 100.2 kA/m. In contrast to the coercivity, the remanence varied significantly depending on the region. The difference in remanence between regions 2 and 3 was approximately 0.30 T. During the die-upset process, the remanence was expected to decrease the most in region 2, where the corners were located, because more stress was concentrated in this area than in other regions [18,19]. In addition, when a rectangular die is used, a higher stress concentration is expected to occur at the corners and along the shorter edges [18,19]. For the magnets with 2:1 and 1:1 ratios, the differences in coercivity were not significant, and the remanence variation across different regions was relatively small. The remanence differences between regions 2 and 3 for the magnets with 2:1 and 1:1 ratios were 0.08 and 0.12 T, respectively, representing significant improvements compared with the magnet with a 4:1 ratio. As the difference in the length-to-width ratio decreased, the magnetic property differences between the central and corner regions of the magnet decrease. If the die is changed from a rectangular shape to a square shape, the sides become equal in length, increasing the probability that the pressure will be evenly distributed during the die-upset process. This uniform distribution of pressure across the entire magnet can reduce the remanence variation.

**Figure 4:**
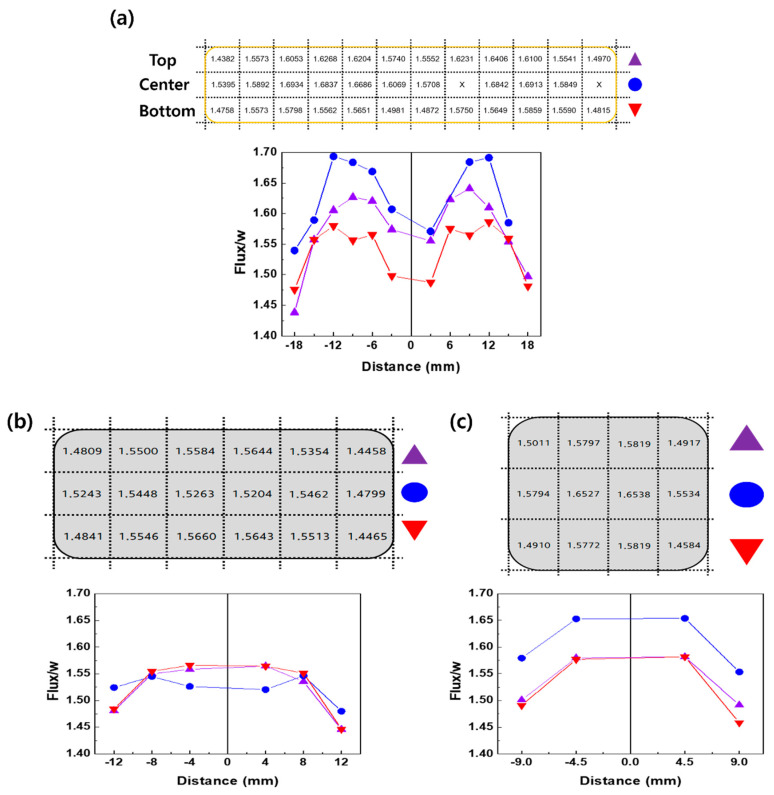
Magnetic flux per unit weight variation in each section along the length and width of the magnet. Schematics of the magnets with (**a**) 4:1, (**b**) 2:1, and (**c**) 1:1 ratios are presented, showing the flux per unit weight values at different positions.

Figure 4 shows the variations in the magnetic flux per unit weight according to the positional differences for the magnets with ratios of 4:1, 2:1, and 1:1. For each magnet, the top, center, and bottom sections were divided along the width and further subdivided into equal lengths along the length to analyze the magnetic flux at different positions. Among these, the center section of the magnet with a 4:1 ratio exhibited the highest magnetic flux per unit weight, as shown in Figure 4a. The magnetic flux per unit weight was lower in the top and bottom sections than in the center section. Along the length, the flux per unit weight values was measured at 3 mm intervals, and the results indicated that the flux per unit weight increased up to approximately ±12 mm from the center, but decreased beyond ±12 mm. The difference between the maximum and minimum values of the magnetic flux per unit weight exceeded 0.25. For the magnet with a reduced length-to-width ratio of 2:1, the variation in the magnetic flux per unit weight across the top, center, and bottom sections decreased compared to the magnet with a 4:1 ratio (Figure 4b). Along the length, there was little to no variation in magnetic flux per unit weight from 0 to ±8 mm, and while relatively low magnetic flux per unit weight values were observed at ±12 mm, the differences were not significant. For the magnet with a 1:1 ratio, the highest flux per unit weight was observed in the center section, as for the magnet with a 4:1 ratio. However, the difference in the flux between the top and bottom positions was far smaller than that for the magnet with a 4:1 ratio. As the length-to-width ratio changed from 4:1 (Figure 4a) to 1:1 (Figure 4b), the differences in flux at the surfaces in contact with the die and at the corner positions decreased, and the uniformity improved. In the aforementioned study by Dirba et al., crack-free die-upset magnets with a radial shape were manufactured [16]. Because of the symmetry of the circle, the force was evenly distributed around the entire perimeter, resulting in magnets with a uniform texture. However, in this case, the magnets were produced in a rectangular shape (with 4:1 and 2:1 ratios) or square shape (with a 1:1 ratio), leading to an uneven distribution of the force applied to the material during the die-upset process, particularly at the corners [18,19]. Thus, the rectangular and square shapes are more prone to experiencing stress concentration at the corners.

**Figure 5:**
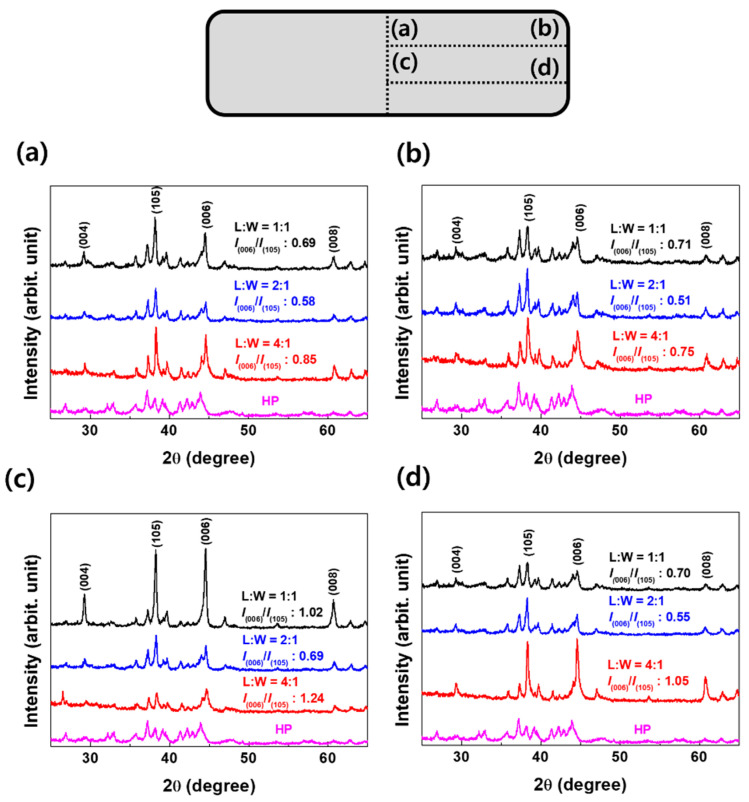
XRD patterns of an Nd–Fe–B HP magnet and die-upset magnets subjected to various conditions. Refer to the simplified illustration of a magnet above the graph for positions (**a**–**d**). R represents the ratio of the XRD peak intensities at (006)/(105).

Figure 5 shows the XRD patterns at different positions of the HP and die-upset magnets with 4:1, 2:1, and 1:1 ratios. The main diffraction peaks of all magnets correspond to the Nd_2_Fe_14_B (2:14:1) phase, indicating that the Nd_2_Fe_14_B phase with tetragonal structure (space group P42/mnm) had been formed in all magnets. Among these, the (004), (006), and (008) peaks, which indicate the c-axis of direction, are displayed, along with the (105) peak, which is not along the c-axis. The peaks that are not displayed also correspond to the 2:14:1 phase. The XRD pattern of the HP magnet indicated an isotropic structure. In contrast to the HP magnets, the grain alignment of the die-upset magnets exhibited growth along the (00L) direction. It is well known that the grains in HP magnets are generally isotropic or randomly oriented. Because the grains did not undergo significant deformation, their orientation remained mostly unchanged during the hot-press process. The orientation ratio (R) *I*_(006)_/*I*_(105)_ for each magnet position is shown in Figure 5. This parameter is commonly used to characterize the degree of texture resulting from the deformation of Nd_2_Fe_14_B grains, where all the stacked platelet-shaped grains rotate their c-axis parallel to the deformation direction [20,21]. The R values varied significantly among the different regions for all the die-upset magnets, as shown in Figure 5. Specifically, the (00L) grain alignment of the magnet with the 4:1 ratio varied significantly depending on the position. Region 3—the central part of the magnet with a 4:1 ratio—exhibited an R-value of 1.24, whereas region 2, which was located at the corner, exhibited an R-value of 0.75. This was consistent with the remanence differences shown in Figure 3. In contrast, the magnet with the 2:1 ratio exhibited a uniform texture across all regions, but the R value did not exceed 1 at any position, as shown in Figure 5b. The (00L) grain alignment of the magnet with the 2:1 ratio was not well developed in any region. Additionally, as shown in Figure 4, the flux values at all positions are lower than those of the 4:1 and 1:1 ratio magnets, which was consistent with the XRD results. For the magnet with a 1:1 ratio, the R value exceeded 1 in region 3, i.e., the center of the magnet. This magnet exhibited higher R values than the 2:1 ratio magnet in all regions. As the length-to-width ratio changed from 2:1 to 1:1, making the width and length equal, the grain alignment in the (00L) direction improved.

**Figure 6:**
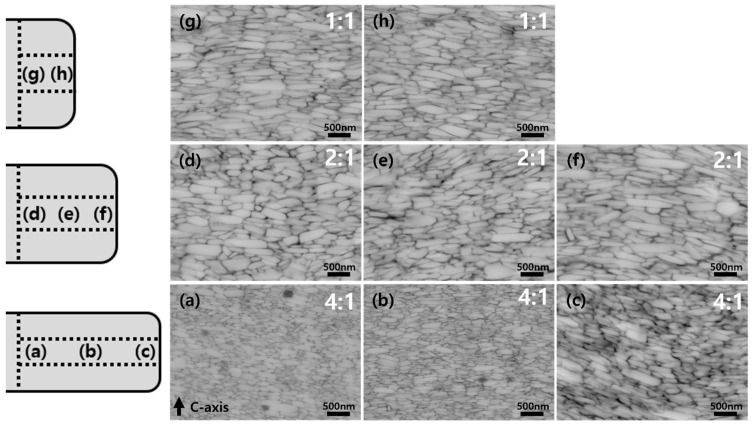
Position-specific BSE images of Nd–Fe–B hot-compaction magnets with length-to-width ratios of (**a**–**c**) 4:1, (**d**–**f**) 2:1, and (**g**,**h**) 1:1. Refer to the simplified illustrations of half of a magnet with 4:1, 2:1, and 1:1 ratios to the left of the images, showing positions from the center to the edge, indicated by letters in each figure.

Figure 6 shows cross-sectional backscattered electron (BSE) SEM images of magnets with 4:1, 2:1, and 1:1 ratios at various positions. The average lateral grain sizes of the magnets with ratios of 4:1, 2:1, and 1:1 are 301 nm, 372 nm, and 403 nm, respectively. The corresponding standard deviations of the grain size distributions are 176 nm, 103 nm, and 80 nm. As the length-to-width ratio changed from 4:1 to 1:1, the average grain size increased, but the distribution improved. The grain size of each magnet was analyzed based on different locations, as shown in Figure 6. The magnet with a 4:1 ratio exhibited significant differences in the size of the platelet-shaped grains depending on the position, as shown in Figure 6a–c. The grain size increased from the center of the magnet toward the edges. The average grain size of the magnet with a 4:1 ratio was 250 nm in Figure 6a, 300 nm in Figure 6b, and 450 nm in Figure 6c along the c-plane (D_c_) of the Nd_2_Fe_14_B phase, and additional growth was observed along the c-axis (D_ab_) of the Nd_2_Fe_14_B phase. During the die-upset process, the applied pressure was not uniform across the magnet and varied depending on its shape and position. In particular, for the rectangular shape, there was a higher degree of stress concentration at the edges than at the center, leading to easier grain deformation and growth at the edges. Additionally, the material flows under pressure, deforming relatively uniformly in the center section, whereas near the edges, the material flow can be restricted or uneven [18,19]. As a result, grain growth was more pronounced at the corners. However, the average grain size of the magnet with a 4:1 ratio was far smaller than those of the magnets with 2:1 and 1:1 ratios. As the grain size decreases, the coercivity of the magnet increases because the movement of the domain walls within the magnet becomes more difficult [5]. The magnet with a 4:1 ratio, characterized by the smallest grains, exhibited the highest coercivity among the magnets. However, the overall reduction in remanence observed in the magnet with a 4:1 ratio can be attributed to several factors. Although the R values were high across all regions in the XRD results, the strong crystallographic texture did not result in improved magnetic properties. This discrepancy can be explained through SEM analysis, which revealed significant differences in grain size and distribution between the center and edges of the magnet with a 4:1 ratio. These variations were presumably caused by stress differences between the center and edge regions, as shown in Figure 6a–c. Furthermore, during processing, the sample may have experienced residual stresses or strain that adversely affected the magnetic properties, despite the favorable crystallographic texture [4]. For the 2:1 magnet, the average grain size along the c-plane of the Nd_2_Fe_14_B phase (perpendicular to the c-axis) was approximately 530 nm (Figure 6d), 545 nm (Figure 6e), and 520 nm (Figure 6f). Although no significant difference in lateral grain size was observed between the center and the area near the corners, misaligned fine nanocrystalline grains with sizes of 100–200 nm were observed instead of plate-like grains growing along the c-plane. Although the lateral grain size at different positions exceeded those of the other magnets, the grains grew uniformly. As seen in Figure 6g,h, the magnet with a 1:1 ratio, which had the highest values of remanence and (*BH*)_max_, exhibited the largest lateral grain size compared to the other magnets, and no misaligned fine nanocrystalline grains were observed. The grain growth was uniform across all regions.

Table 3 presents the key magnetic properties, grain aspect ratios, and orientation deviation of the magnets with length-to-width ratios of 4:1, 2:1, and 1:1. The magnetic properties, including the (*BH*)_max_, grain aspect ratio (*D*_c_/*D*_ab_), and orientation deviation (*D*_od_), are shown. The grain aspect ratio was defined as the ratio of the width to the height of a Nd_2_Fe_14_B grain (*D*_c_/*D*_ab_). The grain aspect ratio (*D*_c_/*D*_ab_), which reflects the ratio of the grain dimensions along the a-b plane relative to the c-axis, decreased from 4.18 for the 4:1 ratio magnet to 3.98 for the 2:1 ratio magnet. Interestingly, the *D*_c_/*D*_ab_ ratio increased to 4.36 for the 1:1 ratio magnet. The analysis results show that the aspect ratio is not significantly related to the length-to-width variation of the magnet. X. Xia et al. reported that the grain aspect ratio and grain orientation deviation are not directly related in a simple cause-and-effect manner. While increasing the Nd–Cu–Al content affects both the aspect ratio and orientation, these changes occur independently of each other [22,23]. Therefore, in this study, the analysis focused on the grain orientation perspective when examining changes in the length-to-width ratio of the magnet. The grain orientation degree was analyzed based on the misalignment angle of the c-axis of the Nd_2_Fe_14_B grains. The magnet with a 4:1 ratio exhibited a significant deviation of −7.47°, indicating a higher degree of grain misalignment. As the length-to-width ratio decreased, the *D*_od_ value improved, with the 2:1 magnet exhibiting a deviation of −2.08°. The magnet with a 1:1 ratio exhibited the smallest deviation of 0.59°, indicating that the grains were better aligned in this configuration. Overall, these results indicated that the grain orientation and (*BH*)_max_ of the magnets were significantly affected by the length-to-width ratio. Although the grain size was the largest in the magnet with a 1:1 ratio, leading to a slight reduction in coercivity, the more anisotropic nature of the magnet with a 1:1 ratio, as indicated by the *D*_od_ values, contributed to its superior (*BH*)_max_ and overall magnetic performance compared with magnets with higher length-to-width ratios.

## 4. Conclusions

We investigated the effects of the length-to-width ratio on the magnetic and microstructural properties of die-upset Nd–Fe–B magnets. In particular, we developed a die-upset magnet with a uniform shape and no significant cracking. As the length and width of the magnet became closer, the lateral grain size increased, slightly reducing the coercivity; however, it was possible to produce a magnet with a uniform grain-size distribution. As the length-to-width ratio decreased from 4:1 to 1:1, the coercivity decreased slightly from 1090 to 1030 kA/m, while the remanence increased significantly from 1.14 to 1.21 T. As the pressed surface changed from rectangular to square, stress was applied more uniformly at both the center and corners, leading to improved consistency in the magnetic properties across different positions. Therefore, compared to the magnet with a 4:1 ratio, an 11.9% enhancement in (*BH*)_max_ (285.6 kJ/m^3^) was achieved for the die-upset magnet with a length-to-width ratio of 1:1, owing to the uniformity of the grain size and improved grain alignment. These findings have practical implications for the production of high-performance Nd–Fe–B hot-deformed magnets in applications such as EV/HEV traction motors, where uniform magnetic properties are essential. Furthermore, the improvements observed with the 1:1 ratio suggest that optimizing the shape and dimensions of die-upset magnets could lead to further enhancements in their magnetic performance. Future studies could focus on refining the processing techniques and exploring alternative compositions to further improve the coercivity and overall efficiency of these magnets.

## Figures and Tables

**Table 1:** Dimensions (length, width, and thickness) of Nd–Fe–B die-upset magnets with length-to-width ratios of 4:1, 2:1, and 1:1.

Ratio (Length–Width)	Length (mm)	Width (mm)	Thickness (mm)
4:1	36	9	2.2
2:1	25.45	12.73	2.2
1:1	18	18	2.2

**Table 2:** Magnetic property differences according to the position for the magnets with 4:1, 2:1, and 1:1 ratios.

Region 1	Magnet with 4:1 Ratio	Magnet with 2:1 Ratio	Magnet with 1:1 Ratio
*B*_r_ (T)	1.05	1.12	1.15
*H*_cj_ (kA/m)	914.3	756.8	688.3
(*BH*)_max_ (kJ/m^3^)	202.9	226.5	243.7
**Region 2**	**Magnet with 4:1 ratio**	**Magnet with 2:1 ratio**	**Magnet with 1:1 ratio**
*B*_r_ (T)	0.92	1.05	1.06
*H*_cj_ (kA/m)	952.5	752.0	747.2
(*BH*)_max_ (kJ/m^3^)	155.8	197.2	202.3
**Region 3**	**Magnet with 4:1 ratio**	**Magnet with 2:1 ratio**	**Magnet with 1:1 ratio**
*B*_r_ (T)	1.22	1.13	1.18
*H*_cj_ (kA/m)	929.5	692.3	736.1
(*BH*)_max_ (kJ/m^3^)	281.6	231.4	258.1
**Region 4**	**Magnet with 4:1 ratio**	**Magnet with 2:1 ratio**	**Magnet with 1:1 ratio**
*B*_r_ (T)	1.04	1.12	1.13
*H*_cj_ (kA/m)	852.3	758.4	694.7
(*BH*)_max_ (kJ/m^3^)	196.0	225.0	233.8

**Table 3:** Maximum energy product ((*BH*)_max_), grain aspect ratio (*D*_c_/*D*_ab_), and orientation deviation (*D*_od_) for magnets with different length-to-width ratios.

	Magnet with 4:1 Ratio	Magnet with 2:1 Ratio	Magnet with 1:1 Ratio
(*BH*)_max_ (kJ/m^3^)	255.3	274.5	285.6
*D*_c_/*D*_ab_	4.18	3.98	4.36
*D*_od_ (°)	−7.47	−2.08	0.59

## Data Availability

The data presented in this research are available on request from the corresponding author (due to privacy).

## References

[1] Hono K., Sepehri-Amin H. (2012). Strategy for high-coercivity Nd–Fe–B magnets. Maters. Scr. Mater..

[2] Jin J.Y., Ma T.Y., Zhang Y.J., Bai G.H., Yan M. (2016). Chemically inhomogeneous RE-Fe-B permanent magnets with high figure of merit: Solution to global rare earth criticality. Sci. Rep..

[3] Bae K.H., Kim T.H., Lee S.R., Kim H.J., Lee M.W., Jang T.S. (2014). Magnetic and microstructural characteristics of DyF_3_/DyH*_x_*dip-coated Nd–Fe–B sintered magnets. J. Alloys Compd..

[4] Hioki K. (2021). High performance hot-deformed Nd-Fe-B magnets. Sci. Technol. Adv. Mater..

[5] Hioki K., Hattori A., Iriyama T. (2014). Development of Dy-free hot-deformed Nd-Fe-B magnets by optimizing chemical composition and microstructure. J. Magn. Soc. Jpn..

[6] Sepehri-Amin H., Ohkubo T., Nishiuchi T. (2010). The mechanism of coercivity enhancement by the grain boundary diffusion process of Nd-Fe-B sintered magnets. Scr. Mater..

[7] Rieger G., Seeger M., Sun L., Kronmüller H. (1995). Micromagnetic analysis applied to melt-spun NdFeB magnets with small additions of Ga and Mo. J. Magn. Magn. Mater..

[8] Seeger M., Köhler D., Kronmüller H.J. (1994). Magnetic and microstructural investigations of melt-spun Fe(NdPr)B. J. Magn. Magn. Mater..

[9] Mishra R.K., Brewer E.G., Lee R.W. (1988). Grain growth and alignment in hot deformed Nd-Fe-B magnets. J. Appl. Phys..

[10] Mishra R.K., Chu T.Y., Rabenberg L.K. (1990). The development of the microstructure of die-upset Nd-Fe-B magnets. J. Magn. Magn. Mater..

[11] Liu J., Sepehri-Amin H., Ohkubo T., Hioki K., Hattori A., Hono K. (2014). Microstructure evolution of hot-deformed Nd-Fe-B anisotropic magnets. J. Appl. Phys..

[12] Cui J., Ormerod J., Parker D.S., Ott R., Palsyuk A., McCall S., Paranthaman M.P., Kesler M.S., McGuire M.A., Nlebedim C.N. (2022). Manufacturing Processes for Permanent Magnets: Part II—Bonding and Emerging Methods. JOM.

[13] El-Moneim A.A., Gutfleisch O., Plotnikov A., Gebert A. (2002). Corrosion behaviour of hot-pressed and die-upset nanocrystalline NdFeB-based magnets. J. Magn. Magn. Mater..

[14] Iwasaki K., Shinoda M., Tanigawa S., Tokunaga M. (1992). Development of trapezoid-shaped Nd–Fe–B die-upset magnets by preform method. IEEE Trans. Magn..

[15] Brown D.N., Smith B., Ma B.M., Campbell P. (2004). The dependence of magnetic properties and hot workability of rare earth-iron-boride magnets upon composition. IEEE Trans. Magn..

[16] Dirba I., Sawatzki S., Gutfleisch O. (2014). Net-shape and crack-free production of Nd–Fe–B magnets by hot deformation. J. Alloys Compd.

[17] Venkitaraman A.K., Kosuru V.S.R. (2023). Trends and challenges in electric vehicle motor drivelines—A review. Int. J. Electr. Comput. Eng. Syst..

[18] Louhghalam A., Igusa T., Park C., Choi S., Kim K. (2011). Analysis of stress concentrations in plates with rectangular openings by a combined conformal mapping—Finite element approach. Int. J. Solids Struct..

[19] Yang Y., Liu J., Cai C. (2008). Analytical solutions to stress concentration problem in plates containing rectangular hole under biaxial tensions. Acta Mech. Solida Sin..

[20] Jang Y.R., Kim W.J., Kim S.M., Lee W. (2024). Effect of pressure on Ce-substituted Nd-Fe-B hot-deformed magnets in the hotpressing process. Materials.

[21] Zhang H., Wang Y., Wang H., Huo D., Tan W. (2022). Room-temperature magnetoresistive and magnetocaloric effect in La1−xBaxMnO3 compounds: Role of Griffiths phase with ferromagnetic metal cluster above Curie temperature. J. Appl. Phys..

[22] Tang X., Li J., Miyazaki Y., Sepehri-Amin H., Ohkubo T., Schrefl T., Hono K. (2020). Relationship between the thermal stability of coercivity and the aspect ratio of grains in Nd-Fe-B magnets: Experimental and numerical approaches. Acta Mater..

[23] Xia X., Liu M., Zhang T., Dong Q., Zhang L., Zhou L., Li M. (2020). Dependence of grain size and aspect ratio on grain boundary additives in hot-deformed Nd-Fe-B magnets. Mater. Charact..

